# GsmPlot: a web server to visualize epigenome data in NCBI

**DOI:** 10.1186/s12859-020-3386-0

**Published:** 2020-02-12

**Authors:** Jia Li, Yue Yin, Mutian Zhang, Jie Cui, Zhenhai Zhang, Zhiyong Zhang, Deqiang Sun

**Affiliations:** 1 Center for Epigenetics & Disease Prevention, Institute of Biosciences and Technology, Texas A&M University College of Medicine, Houston, TX 77030 USA; 2 Key Laboratory of Special Pathogens and Biosafety, Wuhan Institute of Virology, Chinese Academy of Sciences, Wuhan, 430071 China; 3 Center for Bioinformatics, School of Basic Medical Sciences, Southern Medical University, Guangzhou, China; 4 The Third Affiliated Hospital of Guangzhou Medical University, Guangzhou, China

**Keywords:** GsmPlot, Epigenetics, Webserver, NCBI, Visualization

## Abstract

**Background:**

Epigenetic regulation is essential in regulating gene expression across a variety of biological processes. Many high-throughput sequencing technologies have been widely used to generate epigenetic data, such as histone modification, transcription factor binding sites, DNA modifications, chromatin accessibility, and etc. A large scale of epigenetic data is stored in NCBI Gene Expression Omnibus (GEO). However, it is a great challenge to reanalyze these large scale and complex data, especially for researchers who do not specialize in bioinformatics skills or do not have access to expensive computational infrastructure.

**Results:**

GsmPlot can simply accept GSM IDs to automatically download NCBI data or can accept user’s private bigwig files as input to plot the concerned data on promoters, exons or any other user-defined genome locations and generate UCSC visualization tracks. By linking public data repository and private data, GsmPlot can spark data-driven ideas and hence promote the epigenetic research.

**Conclusions:**

GsmPlot web server allows convenient visualization and efficient exploration of any NCBI epigenetic data in any genomic region without need of any bioinformatics skills or special computing resources. GsmPlot is freely available at https://gsmplot.deqiangsun.org/.

## Key points

- The public epigenetic data stored in NCBI is essential for biomedical research but an easy-to-use tool with the quick visualization function is missing.
- We present GsmPlot, a user-friendly web server to allow scientists without any bioinformatics expertise, or any high-performance computational resources to easily visualize public epigenetic data in NCBI.
- GsmPlot can be used to study the crosstalk between histones, DNA modifications, co-binding of TFs, and other epigenetic factors at any functional genomic regions or user defined regions.
- GsmPlot supports user-server interactions which allow users to choose their concerned regions to further explore different epigenetic factor interactions among multiple samples

## Background

Epigenetic mechanisms alter phenotypes by regulating gene expression patterns without altering the DNA sequences in response to physiological or pathological signals [1]. Due to the technology advances of high-throughput sequencing, such as chromatin immunoprecipitation sequencing (ChIP-seq), whole genome-wide sodium bisulfite sequencing (WGBS) [2], anti-CMS immunoprecipitation (CMS-IP)-seq [3], and ATAC-seq [4], an extremely large amount of epigenomic data has been generated and published. Epigenetic factors including histone modifications, TFs bindings, DNA modifications and chromatin accessibilities, are always dynamically interact with each other to shape the epigenomic landscape specifically to certain biological process [5–8]. Therefore, it is important to compare different epigenetic factors visually from different studies (public data) to ensure a properly comprehensively interpretation. NCBI Gene Expression Omnibus [9, 10] is a primary data source for high-throughput sequencing data repository, which includes epigenetic data generated from various species, cell types, diseases and experimental conditions. In GEO, every dataset has multiple GSM IDs, each of which corresponds to one raw sequencing file in Fastq format and processed file in formats such as Wig, BigWig and BedGraph. BigWig files are binary and indexed files containing genome wide data signals at various resolutions [11], and are easier to manipulate compared with Wig and BedGraph.

Although DaVIE [12], Octopus-toolkit [13] and EpiMINE [14] provide visualization of public data, they require installation of some necessary software to user’s computer, require extensive knowledge of the pipeline from researchers to run the software and analyze the epigenetic data, and require a good computation capacity. Both WashU epigenome browser [15] and UCSC genome browser [16] are excellent epigenome data browsers, which allow users to upload bigwig files to visualize. However, users are required to set up public URLs for their data which requires bioinformatic expertise and usually a webserver owned by the user. Many researchers in the biomedical field do not have bioinformatics expertise or high-performance computer resources to analyze, reform and visualize the public data. Currently, there is no user-friendly tool with convenient visualization function that do not require any complicated installation step or any computational skills or infrastructure for next-generation sequencing data in NCBI.

To alleviate these limitations, we developed GsmPlot, a user-friendly web server to easily generate customized visualizations for the public data in GEO and additionally provide interactive explorations. GsmPlot is convenient to use as it need only GSM IDs or the bigwig files provided by user. GsmPlot can conveniently generate profile plots on functional genome elements (gene, promoter, exon, intron, or any regions defined by user) or visualization on one specifically concerned region through UCSC genome browser integration. Moreover, GsmPlot allows interactive selection of regions with specific epigenetic patterns in the heatmap for further explorative study.

## Results

GsmPlot provides two flexible methods for the user to query the data: GSM IDs or bigwig files on user computer. GsmPlot automatically downloads the bigwig/wig/bedgraph file from GEO or from the user computer to the web server. Users can profile the data along user-defined genome intervals by providing BED files or along user-defined gene sets by providing gene names (Additional file 1: Figure S1). There is no limit on the number of GSM IDs or number of BigWig files, meaning GsmPlot can easily draw RNA-Seq, ChIP-Seq, ATAC-Seq, Bis-Seq or any other type of sequencing data altogether in one plot. We found that more than 65% of ChIP-seq, ATAC-seq and Bisulfite-seq datasets stored in GEO have bigwig, wig or bedgraph files available (Additional file 6: Table S1), making GsmPlot a significant tool to revisit these large number of datasets in NCBI. Moreover, GsmPlot can automatically perform reference genome sanity check, and lift over genome versions whenever necessary to correctly utilize all the data stored in NCBI for the past decades with different genome versions. With the same datasets and same plot setting, GsmPlot is relatively fast in our tests for typical datasets in GEO (Additional file 6: Table S2, S3).

Furthermore, GsmPlot embedded the public DNA methylation (5mC) and hydroxymethylation (5hmC) data for human and mouse ES cells [17–19] . Therefore, researchers can visualize the 5mC or 5hmC distribution on concerned transcription factor (TF) binding regions, histone modification regions, or any other concerned regions, looking for clues about how DNA modification interacts with TFs, histones, and so on. In addition, co-binding of TFs is an important gene regulatory mechanism [20]. GsmPlot can also be used to study the co-binding of two or more TFs by integrating the public ChIP-seq data (such as Cistrome [21] and ENCODE database) and the user-provided ChIP-seq data. Such integration of DNA methylation, hydroxymethylation, and TF binding data is extremely useful in terms of interpreting the regulation functions of epigenetic factors. Most importantly, GsmPlot integrated the UCSC genome browser visualization at the end of the analysis pipeline so users can browse to specific genomic locations to visualize these data signals.

**Fig. 1:**
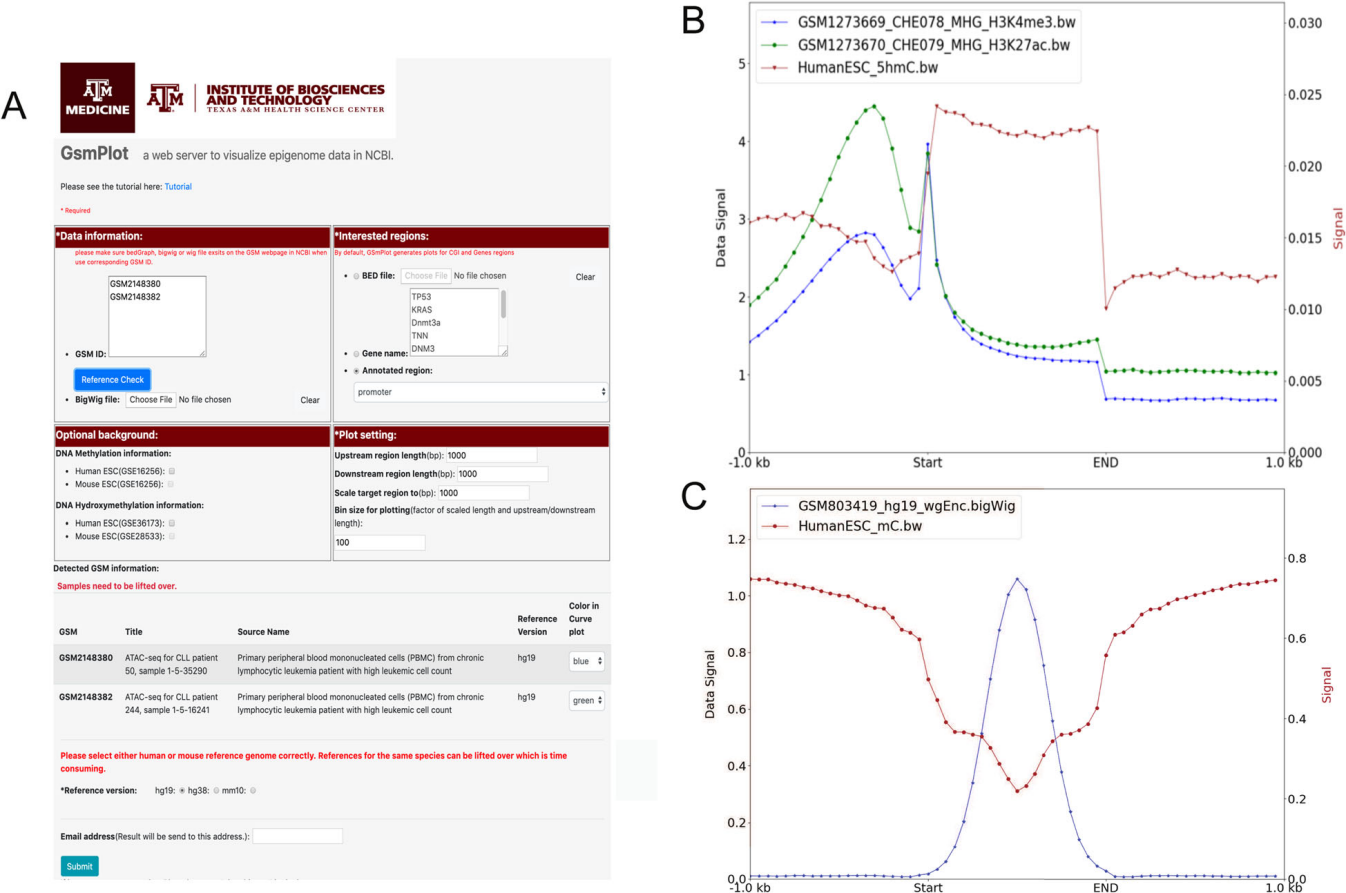
**a** GsmPlot website interface. **b** Average H3K27Ac (blue curve), H3K4me3 (red curve), and 5hmC (green curve) ChIP-Seq signals along genes. **c** Average CTCF ChIP-Seq signal and DNA methylation Bis-Seq signal along CTCF binding sites

Figure 1a shows an example using GsmPlot to investigate the crosstalk between histone modification and DNA methylation. We entered GSM1273669 (H3K4me3 ChIP-Seq) and GSM1273670 (H3K27ac ChIP-Seq) in the “Data information” box and selected “Human ESC” for 5hmC information. We optionally plot the 1000 bases upstream and downstream of the selected regions, and scale all target regions to be 1000 bases. We also set the bin size to be 50 bases to get high-resolution curves. In the result, the blue and green curves in Fig. 1b indicated that the average signal of H3K4me3 and H3K27ac are highly enriched around promoter regions with double peaks, consistent with a previous study [22] and the 5hmC signal is enriched in genebody regions. In an example region shown in the UCSC genome browser in Additional file 2: Figure S2, the H3K4me3 and H3K27Ac peaks are well aligned with gene promoters. This example confirmed that our program is correct and efficient.

GsmPlot also can be used to investigate the relationship between TFs and DNA methylation or hydroxymethylation. Figure 1c shows that the CTCF binding regions in hESC downloaded from GSM803419 generally have a depletion of 5mC but accompanied with complex DNA 5hmC distribution (Additional file 3: Figure S3A). In the center of the CTCF peak regions, we could observe depletion of 5mC signal (Additional file 3: Figure S3B). This result is also consistent with a previous study [23], proving again that GsmPlot can process and plot multiple signals correctly.

**Fig. 2:**
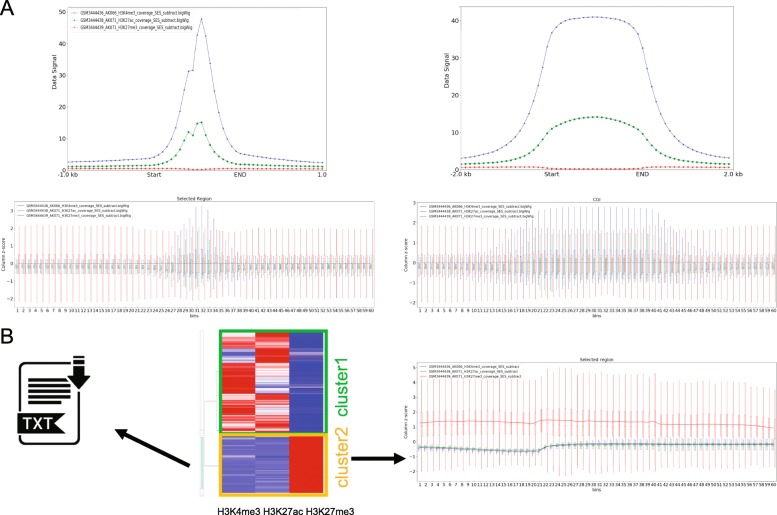
**a** GsmPlot default figures for the average signal curve (upper) and the z-score boxplots (lower) along TSS (left) and CpG Island (right) regions. Blue: H3K4me3; Green: H3K27ac; Red: H3K27me3. **b** GsmPlot interactive heatmap allowing users to choose specific regions to dynamically plot column z-score boxplot and download the selections

Epigenetic data from different sources are usually generated and normalized differently, preventing such data to be compared directly. To circumvent this problem, we can use z-score to replace raw wig signal to allow direct comparison. For each sample, we calculate the average bigwig signal in bins of user-defined size along concerned regions. Then, we calculate z-scores of the corresponding wig values for each bin in each region (Additional file 4: Figure S4). In the example illustrated by Fig. 2a and Additional file 5: Figure S5, we plotted the aggregated profiles on the upper panel and the z-score boxplots on the lower panel for H3K4me3, H3K27ac and H3K27me3 (GSM3444436, GSM3444438 and GSM3444439) in glioblastoma tissue. From both the average wig profiles and the z-score boxplots, we could clearly see the enrichment of H3K4me3 and H3K27Ac but not H3K27me3 on the selected TSS and CGI regions, and no enrichment on the genebody regions. Furthermore, as a unique feature of GsmPlot, we developed an interactive heatmap to aid users to explore the potentially interesting regions enriched with epigenetic factors. We choose the top 5 k (by default) most variable regions among all samples to plot heatmap (Fig. 2b). Cluster 1 represents active genes with both H3K4me3 and H3K27ac enriched in promoter and cluster 2 represents repressed genes with H3K27me3 enriched in promoter. Users can slide the side bar of heatmap to select the regions with specific patterns. The z-score boxplot for these selected regions will be re-plotted. And the genomic locations of these selected regions can be downloaded as text file for further study. For example, users can upload this file to GsmPlot as concerned regions to investigate how epigenetic factors distribute on this specific set of regions.

**Fig. 3:**
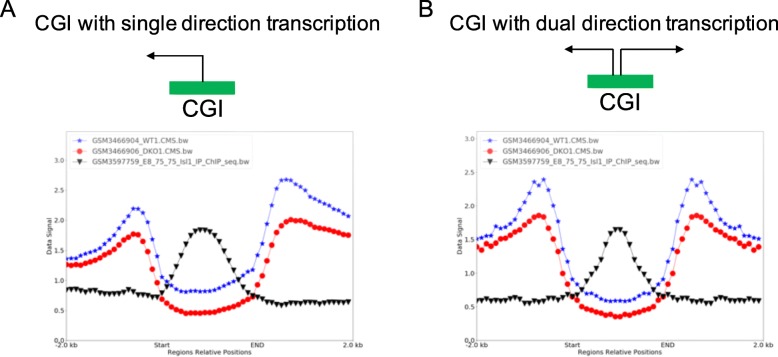
**a** 5hmC signal distribution around CGIs with single transcriptional direction; **b** 5hmC signal distribution around CGIs with dual transcriptional directions. Blue: 5hmC signal from WT mouse heart; red: 5hmC from Tet2/3 knockout mouse heart; black: mouse heart *Isl1* ChIP-seq signal

As an example, to illustrate that GsmPlot has the potential to shape novel biological hypothesis or discoveries, we explored the potential roles of DNA hydroxymethylation (5hmC) around CGI regions in heart development. We used mouse heart DNA hydroxymethylation data (CMS-IP) from wildtype (GSM3466904) and Tet2/3 knockout (GSM3466906) mice [24]. We also included mouse heart ChIP-seq (GSM3597759) data for *Isl1*, which is a cardiac progenitor marker gene, and is important for heart development [25, 26]. Our GsmPlot results showed that around CGIs with single transcriptional direction, 5hmC exhibits unbalanced and directional distribution pattern (Fig. 3a). On the contrary, 5hmC level is symmetric on upstream and downstream of CGIs with dual transcriptional directions. Dramatically decreased 5hmC level in Tet2/3 KO mouse hearts are observed in both CGIs with single and dual transcriptional directions (Fig. 3b). Moreover, *Isl1* binding intensity is higher at CGIs with single transcriptional direction than dual directional transcriptions (dash green line). These results indicate that 5hmC may play different roles in terms of how heart related TFs bind to CGIs with single or dual transcriptional directions.

## Discussion

Biomedical data stored in NCBI is valuable for biomedical researchers. However, most researchers and physicians do not have computation skills or infrastructure, and hence this “treasure” could not be used immediately. Even for bioinformaticians, complicated procedures including download, computation, aggregation, hosting of data are required to visualize NCBI data. We developed a web server, GsmPlot, which can download, compute, visualize and compare data. The most important feature of GsmPlot is the ability of performing multiple omics integration studies, such as RNA-seq, Bis-seq, ChIP-seq, ATAC-seq with simply GSM IDs from NCBI. Private data sequenced by users in proper visualization format can be fed into GsmPlot to compare with public data. Compared with other good epigenome analysis platforms, such as EpiMINE, GsmPlot have many advantages. GsmPlot does not need users to download public data manually. GsmPlot do not depend on users’ computer capacity especially for computation intensive bis-seq data, which cannot be handled on a desktop computer. In addition, installation problems, such as software compatibility, software version, could be a big headache for many researchers, but can be completely avoided using GsmPlot. Moreover, interesting regions with certain epigenetic features can be extracted using interactive heatmap, which can be fed into GsmPlot again to explore if there are new epigenetic factors in these interesting regions. Importantly, we have successfully proved GsmPlot’s reliability and its potential ability of making novel biological ideas from three case studies. Above all, GsmPlot is a user-friendly and reliable tool to investigate public epigenetic data, especially for those biomedical researchers who do not have any computation skills.

Although GsmPlot has an email alert for those large data tasks, GsmPlot will add more CPUs to further improve the speed of calculation in the future depending on the demand. The figure’s format, label sizes and other features will be added as user options which will allow users to generate publication quality figures using GsmPlot.

## Conclusions

We have presented GsmPlot, a user-friendly web server for quick visualization and exploration of public NCBI data. To our best knowledge, this is the first webserver that can automatically download data from GEO, transform data, generate images, and support user interaction. Users can easily and quickly visualize and explore any public epigenetic data without requiring of any special training or computing resources, and hence can study the epigenetic mechanism efficiently. The three applications presented above confirmed that GsmPlot can be a huge driver to accelerate the research process by providing convenient visualization of both public and private data, and hence promoting data driven ideas. GsmPlot will dramatically improve the efficiency of utilization of public epigenetic data and further promote the research in epigenetic community.

## Implementation and methods

### Components of GsmPlot

GsmPlot server is composed of three parts: web crawler, data process and web interface. (1). Web crawler was coded in Python 3.5 and specifically designed for NCBI to automatically detect the URLs and download files with bigwig, wig and BedGraph format. We also include genome reference version check in web crawler. Data process include two parts: calculation and visualizations. (2). For data calculation, we wrapped **deepTools** [27] to calculate the average bigwig signal in bins of user-defined size along concerned regions. A matrix of average bigwig signal with **rows as regions** and **columns as bins** are generated, and the column mean values are plotted as aggregated profile. By transforming the wig signal to z-score, we also plot all the z-scores in one bin as a boxplot and so for all bins, as illustrated by the Additional file 4: Figure S4. For the z-score matrix, based on each row’s z-score standard deviation, the top 5 k most variable regions among all samples were chosen to plot heatmap. Users can choose regions based on the heatmap patterns to replot and download the selected regions to do further study. For data visualization, we use in-house scripts coded by Python 3.5 (Matplotlib, https://matplotlib.org/) and R (https://www.r-project.org/). (3). GsmPlot web interface is implemented using HTML, CSS (bootstrap, http://getbootstrap.com/2.3.2/), and JavaScript. The backend of GsmPlot is based on Django web framework (https://www.djangoproject.com/). The interactive functions between users and GsmPlot web server are implemented using jQuery (https://jquery.com). For large data which takes long time to finish the calculation, we include an email alert function by using django.cor.mail function. Due to the limited computing resources, we currently only allow one task for each user at a time. GsmPlot has been tested in Firefox, Chrome, Safari, and Edge.

### Flowchart of GsmPlot

The flowchart of GsmPlot is in Additional file 1: Figure S1. GsmPlot web server friendly accepts GSM IDs or user uploaded bigwig files as input. If the input is a GSM ID, web crawler will search NCBI web sites to locate bigwig files and automatically download the files. At the same time, web crawler will also try to collect the genome reference version information to double check user input information. If the file format is Wig or BedGraph, GsmPlot will automatically transform them to BigWig format. After downloading the files, wrapped deepTools will calculate the average signals on user provided genome regions according to user provided bin size. The downloaded files will be stored in GsmPlot server for 72 h from last access, which will save the downloading time when users reuse this data frequently. If the input files are uploaded by users, GsmPlot will directly proceed to calculation and visualization. “Reference check” function will aid users to choose the right reference version by collecting the reference information from NCBI website. Users can select regions with specific epigenetic patterns in the heatmap. Genomic coordinates of these selected regions can be downloaded in text format which could be further studied.

#### Availability and requirements

**Project name:** GsmPlot.

**Project home page:** https://gsmplot.deqiangsun.org/

**Operating System:** Platform independent.

**Programming language:** Python.

**License:** GNU GPL.

**Other requirements:** Internet Explorer 10 or later.

Discussion Group: https://groups.google.com/d/forum/moabs_msuite

Support email: moabs_msuite@googlegroups.com.

Any restrictions to use by non-academics: None.

## Supplementary information

**Additional file 1: Figure S1**. Scheme for the structure of GsmPlot web server.

**Additional file 2: Figure S2**. Illustration of the data matrix for the profile curve and the z-score boxplots (left), and illustration of the data matrix for the heatmap (right).

**Additional file 3: Figure S3**. UCSC genome browser visualization for RNA-Seq, H3K27Ac, H3K4me3 on an example region for human H1 ESC.

**Additional file 4: Figure S4**. A, the average ChIP-Seq signal along the CTCF binding sites with red curve for the CTCF signal and blue curve for the 5hmc signal. B, The UCSC genome browser visualization for CTCF peak, DNA methylation and DNA hydroxymethylation on an example region. The yellow highlight areas showed the depletion of 5mC at the center of the CTCF peak.

**Additional file 5: Figure S5**. GsmPlot default figures for the average signal curve along the gene body regions. Blue: H3K4me3; Green: H3K27ac; Red: H3K27me3.

**Additional file 6: Table S1**. Statistics for datasets with bigwig/wig/bedgraph format available in GEO. **Table S2**. Processing time for variable files sizes. **Table S3**. Processing time of GsmPlot and EpiMINE.

## Data Availability

The dataset used in this study include the following GSM IDs: Figure 1 B. H3K4me3 and H3K27ac are highly enriched around promoter regions with double peaks. GSM1273669. GSM1273670. Figure 1 C. The CTCF binding regions in hESC generally have a depletion of 5mC but accompanied with complex DNA 5hmC distribution: GSM803419. Figure 2 A. Histone markers (H3K4me3, H3K27ac, H3K27me3) distribution in glioblastoma tissue. GSM3444436. GSM3444438. GSM3444439. Figure 3A, B. potential roles of DNA hydroxymethylation (5hmC) around CGI regions in heart development: GSM3466904. GSM3466906. GSM3597759.

## Abbreviations

ATAC-Seq: Assay for Transposase-Accessible Chromatin using sequencing
Bis-Seq: Bisulfite sequencing
CGI: CpG Island
ChIP-Seq: Chromatin immunoprecipitation sequencing
GEO: Gene Expression Omnibus
GSM: Gene Sample accessions numbers
H3K27ac: Acetylation at the 27th lysine residue of the histone H3 protein
H3K4me3: Addition of three methyl groups to the lysine 4 on the histone H3 protein
NCBI: National Center for Biotechnology Information
RNA-Seq: RNA Sequencing
TF: Transcription Factor
TSS: Transcriptional Start Site
