# Supplementary material for: GsmPlot: a web server to visualize epigenome data in NCBI
Source: BMC Bioinformatics. 2020 Feb 12;21:55. doi: 10.1186/s12859-020-3386-0 (PMC7017537; doi:10.1186/s12859-020-3386-0)
Supplement: Supplementary file 6 — Additional file 6: Table S1. Statistics for datasets with bigwig/wig/bedgraph format available in GEO. Table S2. Processing time for variable files sizes. Table S3. Processing time of GsmPlot and EpiMINE. [file 12859_2020_3386_MOESM6_ESM.docx]

Table S1. Statistics for datasets with bigwig/wig/bedgraph format available in GEO

| Organism | Method | Total datasets | GsmPlot usable  datasets | Ratio |
| --- | --- | --- | --- | --- |
| Human | ATAC-seq | 4,010 | 1,759 | 43.87% |
|  | ChIP-seq | 20,640 | 17,390 | 84.25% |
|  | Bisulfite-seq | 521 | 351 | 67.37% |
|  | Total | 25,171 | 19,500 | 65.16% |
| Mouse | ATAC-seq | 2,921 | 1,677 | 57.41% |
|  | ChIP-seq | 11,666 | 8,540 | 73.20% |
|  | Bisulfite-seq | 552 | 359 | 65.04% |
|  | Total | 15,139 | 10,576 | 65.22% |

Table S2. Processing time for variable files sizes

| Default plot setting | | | |
| --- | --- | --- | --- |
| Number of Files | Average file size (Mb) | Total Processing Time | GSM IDs |
| 1 | 463 | 2 m 30 s | GSM2535467 |
| 1 | 917 | 5 m | GSM2535465 |
| 2 | 350 | 8 m | GSM935297; GSM935299 |
| 2 | 500 | 8 m 25 s | GSM935305; GSM935282 |
| 3 | 433 | 9 m 21 s | GSM935282; GSM935298; GSM935301 |
| 3 | 225 | 11 m 21 s | GSM3073977; GSM3073950; GSM3073962 |
| 4 | 160 | 14 m 24 s | GSM3444438; GSM3444440; GSM3444437; GSM3444439 |
| 5 | 552 | 24 m 28 s | GSM2781481; GSM2535467; GSM2535470; GSM2535468; GSM2535464 |
| 6 | 120 | 20 m 53 s | GSM3073980; GSM3073953; GSM3073981; GSM3073954; GSM3073984; GSM3073966 |

Table S3. Processing time of GsmPlot and EpiMINE

| Reads_number | 400 k | 2,000 k | 4,000 k | 8,000 k | 9,671 k |
| --- | --- | --- | --- | --- | --- |
| Wig file size (MB) | 32 | 115 | 195 | 332 | 353 |
| Bam file size (MB) | 23 | 109 | 214 | 419 | 440 |
| GsmPlot_ | 2 m 31 s | 2 m 57 s | 3 m 10 s | 3 m 26 s | 3 m 30 s |
| EpiMINE_ | 7 m 39 s | 7 m 55 s | 10 m 20 s | 15 m 3 s | 15 m 43 s |
